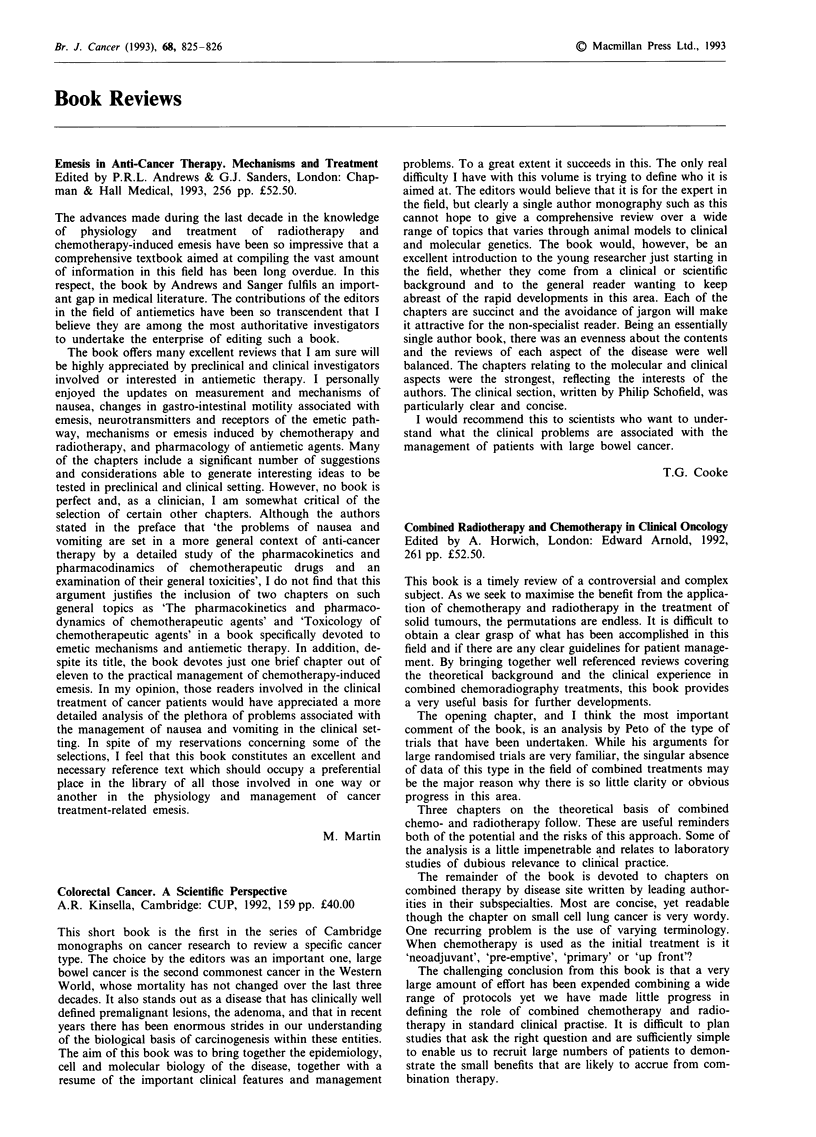# Colorectal Cancer, A Scientific Perspective

**Published:** 1993-10

**Authors:** T.G. Cooke


**Colorectal Cancer. A Scientific Perspective**
A.R. Kinsella, Cambridge: CUP, 1992, 159 pp. £40.00

This short book is the first in the series of Cambridge monographs on cancer research to review a specific cancer type. The choice by the editors was an important one, large bowel cancer is the second commonest cancer in the Western World, whose mortality has not changed over the last three decades. It also stands out as a disease that has clinically well defined premalignant lesions, the adenoma, and that in recent years there has been enormous strides in our understanding of the biological basis of carcinogenesis within these entities. The aim of this book was to bring together the epidemiology, cell and molecular biology of the disease, together with a resume of the important clinical features and management problems. To a great extent it succeeds in this. The only real difficulty I have with this volume is trying to define who it is aimed at. The editors would believe that it is for the expert in the field, but clearly a single author monography such as this cannot hope to give a comprehensive review over a wide range of topics that varies through animal models to clinical and molecular genetics. The book would, however, be an excellent introduction to the young researcher just starting in the field, whether they come from a clinical or scientific background and to the general reader wanting to keep abreast of the rapid developments in this area. Each of the chapters are succinct and the avoidance of jargon will make it attractive for the non-specialist reader. Being an essentially single author book, there was an evenness about the contents and the reviews of each aspect of the disease were well balanced. The chapters relating to the molecular and clinical aspects were the strongest, reflecting the interests of the authors. The clinical section, written by Philip Schofield, was particularly clear and concise.

I would recommend this to scientists who want to understand what the clinical problems are associated with the management of patients with large bowel cancer.

T.G. Cooke